# Clinical Outcome of CT-Guided Iodine-125 Radioactive Seed Implantation for Intrahepatic Recurrent Hepatocellular Carcinoma: A Retrospective, Multicenter Study

**DOI:** 10.3389/fonc.2022.819934

**Published:** 2022-04-08

**Authors:** Qianqian Yuan, Yanli Ma, Linlin Wu, Yuqing Song, Chuang He, Xuequan Huang, Chongshuang Yang, Bin Liu, Hongmei Han, Kaixian Zhang, Junjie Wang

**Affiliations:** ^1^ Department of Oncology, Tengzhou Central People’s Hospital, Zaozhuang, China; ^2^ Department of Oncology, Staff Hospital of Chengde Iron and Steel Group Co. Ltd., Chengde, China; ^3^ Department of Nuclear Medicine, Southwest Hospital, Army Medical University, Chongqing, China; ^4^ Department of Surgery, Tengzhou Central People’s Hospital, Zaozhuang, China; ^5^ Department of Radiation Oncology, The First People’s Hospital of Keerqin District, Tongliao, China; ^6^ Department of Radiation Oncology, Peking University Third Hospital, Beijing, China

**Keywords:** recurrent hepatocellular carcinoma, brachytherapy, prognostic factor, radioactive, seed implantation

## Abstract

The efficacy and safety of CT-Guided Iodine-125 Radioactive Seed Implantation (RSI) for the treatment of intrahepatic recurrent hepatocellular carcinoma (rHCC) were analyzed in this multicenter retrospective study. We reviewed the medical records of patients with rHCC treated with I-125 seed implantation at four different hospitals in China from December 2011 and January 2021. The local progression-free survival (LPFS),liver PFS, and overall survival (OS) were calculated, and the short-term efficacy and treatment-related toxicities were evaluated. A total of 82 patients were enrolled; the median follow-up time was 46 months (range, 3–80 months). The 1-, 3- and 5-year LPFS rates were 63.8%, 27.1%, and 7.9%, respectively, and the corresponding OS rates were 74.8%, 32.9%, and 12.6%, respectively. Univariate analysis showed that factors influencing LPFS included the maximum lesion diameter, Barcelona Clinic Liver Cancer (BCLC) stage, interval between treatment and recurrence, and D90. Multivariate analyses revealed that the BCLC stage, interval between treatment and recurrence, and D90 were independent factors influencing LPFS, whereas BCLC stage, D90, and short-term efficacy were independent factors influencing OS. In summary, I-125 seed implantation is a safe and effective treatment for rHCC. The BCLC stage, interval, and D90 were found to influence the local control. A larger, prospective study is required to confirm the dose-response curve for Iodine-125 RSI of rHCC.

## Introduction

Liver cancer ranked sixth in incidence and fourth in cause of annual cancer-related deaths worldwide in 2018. Hepatocellular carcinoma (HCC) comprises 75%-85% cases of primary liver cancer (1). Intrahepatic tumor recurrent HCC (rHCC) is the major cause of treatment failure in HCC (2, 3). In patients with rHCC, multikinase inhibitors, molecularly targeted therapies, and immune checkpoint inhibitors are administered as second-line systemic therapy (4, 5). Transarterial Chemoembolization (TACE) (2, 6), Transarterial radioembolization (7), radiotherapy (3), and thermal ablation (8) are used as alternative locoregional therapy for rHCC. Iodine-125 radioactive seed implantation (RSI), a minimally invasive internal radiation therapeutic mode, allows continuous irradiation of the tumors after implantation, and the radiation dose of the target tumors is sufficiently high to achieve an ablative effect while sparing the normal tissues. As permanent interstitial brachytherapy, RSI is increasingly used in cancer treatment (9). However, limited studies have investigated the I-125 RSI for recurrent hepatocellular carcinoma. In this retrospective multicenter study, we analyzed the data of patients with rHCC who received I-125 RSI, in order to further assess the safety and efficacy of I-125 RSI for the actual clinical practice of the real world.

## Materials and Methods

### Patients

This was a retrospective study of patients with rHCC at four medical centers in China. We reviewed the medical records of patients treated with CT-guided I-125 RSI between December 2011 and August 2021. The patient inclusion criteria were as follows: (1) Age 18–85 years, with ECOG score ≤2, (2) Confirmed pathological or imaging-based diagnosis (including contrast-enhanced computed tomography(CT), dynamic enhanced magnetic resonance imaging(MRI)) of intrahepatic rHCC (10), (3) Categorized as Child-Pugh class A or B, (4) Tumor size ≤ 7 cm, with lesions ≤3, (5) Intrahepatic tumor(s) recurrences after surgery, ablation, or TACE, (6) The strategic plan for rHCC treatment was discussed by a multidisciplinary cancer team in each hospital, (7) Patients deemed unsuitable for or who refused the molecularly targeted and immune checkpoint inhibitor therapies. Contraindications included: (1) Child-Pugh class C, (2)Tumor size > 7 cm or more than 3 lesions, (3)Bleeding tendency: platelet count ≤50 ×10^9^/L, prothrombin time>18s, prothrombin time activity <40%, (4)Expected survival ≤ 3 months, (5) Tumors located deeply in the liver (large distance from the liver capsule), (6) Tumors at proximity of large vessels.

The patients and their families were informed of the treatment, and subsequently provided written informed consent. The study adhered to the guidelines specified in the Declaration of Helsinki. This study was reviewed and approved by the Ethics Committee of Tengzhou Central People’s Hospital.

### Devices and Instruments

(1) The treatment planning system (TPS) (Beijing University of Aeronautics and Astronautics and Beijing Astro Technology Co. Ltd) was applied to calculate the seed dose distribution of the target tumor and to generate a dose-volume histogram. Planning system source data matched the official, supplementary reports and update of the American Association of Physicists in Medicine (11, 12). (2) Definition AS Bigbore CT, Siemens, Germany. (3) The I-125 seeds were 4.5 mm in length and 0.8 mm in diameter enclosed in a NiTinol capsule (Beijing Atom high Tech Pharmaceutical Company Ltd., China).These seeds could produce 27.4-31.5 keV X-ray and 35.5 keV γ-ray radiations with a half-life of 59.6 days. (4) Radioactive I-125 seed implantation devices (Mick Radio-Nuclear Instruments, Inc., USA and Eckert & Ziegler BEBIG Gmbh.,Germany).

### Preoperative Planning

All patients underwent laboratory testing, including routine blood tests, coagulation function test and liver and kidney function test before the RSI to rule out any contraindications. The patients underwent an enhanced CT scan of 5 mm thickness the week before the treatment. The image data were transmitted to the Treatment Planning System for pre-planning and design. Gross tumor volume (GTV) and adjacent organs at risk (OARs) were identified. And clinical tumor volume(CTV) were calculated by expanding the GTV by 5–6 mm in three dimensions. Next, according to three mutually perpendicular diameters of tumor target volume, the principle of parallel and equidistant source distribution were adopted. Then the implantation design parameters, i.e., prescription dose, seed activity, puncture needle direction, distribution, depth, and seed quantity were determined by TPS. The median prescription dose was 120 Gy (range, 110–160 Gy) and the median activity of the I-125 seed was 0.6 mCi (range, 0.5–0.8 mCi). These patients received the dose of less than 120Gy with high-risk hepatocellular carcinoma less than 10 mm from extrahepatic hollow organs such as stomach and intestines. Others received the dose of 120 to 160Gy.

### Seed Implantation

(1) Free-hand guided I-125 seed implantation: Patients were positioned on the CT-simulator and immobilized with a vacuum pad. After local anesthesia with 1% lidocaine, seed needles were inserted 2–3 cm deep into the target lesion with 0.5–1.0 cm between the two rows using CT guidance. Adjustment of the needle position was performed in real time. A Mick seed implantation gun was used to implant I-125 seeds with a 0.5–1.0 cm distance between two seeds in a receding manner using the applicator. After seed implantation, CT was performed to confirm the seed distribution, and additional seeds were implanted to ensure uniform spatial distribution of the seeds and to minimize the missed area.

(2) Three-dimensional⁃printing coplanar template(3D-PCT) guided I-125 seed implantation: The navigation bracket was connected to the CT carbon fiber bedplate before setting up a patient on the CT-simulator. The 3D⁃PCT was installed in the navigation bracket, and the template was calibrated with laser light and a digital angle meter. Two to three reference needles were placed on the patient’s skin at a predetermined insertion point. CT was performed to observe the needle direction. To calibrate the template to ensure the right direction, all needles were inserted into the target lesion with 0.5–1.0 cm between two rows. Next, the I-125 seed was implanted as described in the free-hand guided procedure.

### Post-Implantation Dose Validation

**Figure 1:**
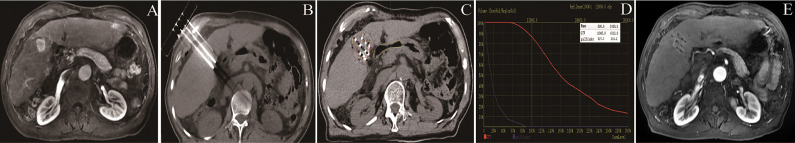
Flow chart showing the seeds implantation procedure: **(A)** Tumor in the right liver; **(B)** 3D⁃printing coplanar template-guided intraoperative needle insertion; **(C)** seeds implantation and postoperative dose verification; **(D)** the dosimetric parameters, e.g. D90, D100 were identified. **(E)** efficacy observation of postoperative follow-up at 6 months.

CT was performed within 1–2 days of the procedure and images were transferred into the TPS for post-plan dose evaluation to check for any seed displacement and to validate the dose. The dosimetric parameters, e.g. D90 (dose delivered to 90% GTV), D100 (dose delivered to 100% GTV), were identified (**Figure 1** showing the seeds implantation procedure).

### Follow-Up and Definitions of Outcomes

Patient vital signs were observed for 24 hour with electrocardiogram monitoring. All postoperative complications were recorded in detail. All patients were followed-up at 4 weeks and 3 months after seed implantation. Thereafter patients were followed-up every three months for two years. At every visit, chest radiography, ultrasonography, blood tests (liver function, serum a-fetoprotein tests), and contrast-enhanced CT or MRI were performed.After two years, follow-up examinations were performed once every six months.

The primary endpoint was local progression-free survival (LPFS). The secondary endpoint was liver progression-free survival (liver PFS) and overall survival (OS). LPFS was defined as the duration between the RSI date and the date of local progression or death. Liver PFS was defined as the duration between the RSI date and the date of intrahepatic recurrence, intrahepatic metastasis or death. OS was defined as the duration between the RSI date and the date of the last follow-up or death. Treatment responses were assessed using the Modified Response Evaluation Criteria in Solid Tumors (mRECIST) (13), as complete response (CR, disappearance of any intratumoral arterial enhancement in intrahepatic target lesions), partial response (PR, at least a 30% decrease in the sum of the longest residual enhancement tumour diameters of the target lesions from baseline), progressive disease (PD, at least a 20% increase and an absolute increase of at least 5 mm in the sum of the longest residual enhancement tumour diameters of the target lesions), or stable disease (SD, between PR and PD). The short-term efficacy was evaluated at 3 months after the implantation. Treatment-related toxicities were evaluated using the Common Terminology Criteria for Adverse Events (CTCAE) (version 5.0) (14) within 3 months after the seed implantation.

### Statistical Analysis

SPSS 26.0 (IBM Corp., Armonk, NY, USA) was used for statistical analysis. Numeration data were expressed as the absolute and/or percentage value. Measurement data were expressed as the median value (range) or mean value ± standard deviation. Chi-square test was used to analyze the tumor response. Survival was estimated using the Kaplan–Meier method, univariate analysis was conducted using the log-rank test, and multifactor analysis was conducted using Cox regression. The results of the multifactor analysis were used to plot nomograms using R (version 4.1.1; http://www.Rproject.org) for visualization. The resolution was expressed as the C-index (concordance index). A C-index of 0.50–0.70 indicated low accuracy, a C-index of 0.71–0.90 indicated good accuracy, and a C-index of 0.91–1.00 indicated high accuracy, similar to that observed for the correlation coefficient. P < 0.05 was considered statistically significant.

## Results

### Patients and Treatment

A total of 82 patients with rHCC met our inclusion criteria and were included in this study; the patients included 69 men (84.1%) and 13 women (15.9%), aged 26–77 years (median age 60). The tumor diameter was 1.2–7.0 cm (median diameter, 4.5 cm), and the total number of intrahepatic lesions targeted was 128 [1 lesion (n = 49), 2 lesions (n = 20), 3 lesions (n = 13)]. Of the included patients, 5 had previously received targeted therapy (sorafenib), 8 had undergone surgery, 25 had received ablation, and 48 had received TACE. Additionally, all patients were ineligible for or had rejected salvage surgery and/or ablation. The interval between the previous treatment and seed implantation was 1–90 months (median, 6 months). The seed number was 11–125 (median, 46) and the postoperative D90 was 86–249 Gy (median, 139 Gy). In total, 76 patients (92.7%) received free-hand guided I-125 seed implantation and 6 (7.3%) received 3D⁃PCT guided I-125 seed implantation (**Table 1**).

**Table 1:** Patient and treatment characteristics (n=82).

Characteristics	(%)	Characteristics	(%)
Age, years,median (range)	60 (26-77)	Maximal tumor diameter, cm, median (range)	4.5 (1.2-7.0)
Sex		AFP level, ng/dl (rang)	15.2 (1.0-6635.0)
Male	69 (84.1)	BCLC stage	
Female	13 (15.9)	A	20 (24.4)
ECOG performance status		B	20 (24.4)
0	42 (51.2)	C	42 (51.2)
1	32 (39.0)	Previous TACE	
2	8 (9.8)	Yes	48 (58.5)
Child-Pugh score		No	34 (41.5)
A	52 (63.4)	Previous Surgery	
B	30 (36.6)	Yes	8 (9.8)
Portal vein thrombosis		No	74 (90.2)
Yes	15 (18.3)	Previous ablation	
No	67 (81.7)	Yes	25 (30.5)
Extrahepatic metastasis		No	57 (69.5)
Yes	30 (36.6)	Previous targeted therapy	
No	52 (63.4)	Yes	5 (6.1)
Treated tumor number		No	77 (93.9)
1	49 (59.8)	Seed implantation method	
2	20 (24.4)	Free-hand guided	76 (92.7)
3	13 (15.9)	3D-printing coplanar template-guided	6 (7.3)

### Tumor Response and Complication

Of the 82 patients included in the study, the short-term efficacy was evaluated at 3 months after the implantation. There were 28 (34.1%) cases of CR, 39 (47.6%) cases of PR, 12 (14.6%) cases of SD, and three (3.7%) cases of PD. The patients were followed up for a median duration of 46 months (range, 3–80 months) until February 2021. Thereafter 51 (62.2%) died, 30 (36.6%) survived, and 1 (1.2%) was lost to follow-up. In total, 13 patients (15.9%) were diagnosed with intrahepatic recurrence, 25 (30.5%) experienced intrahepatic metastasis, and 27 (32.9%) showed extrahepatic distant metastasis.

All patients were allocated the RSI schedule without any interruption caused by treatment-related toxicities. Acute toxicities were observed within 3 months following RSI. The radiation-induced liver disease (RILD) include classic RILD and nonclassic RILD. In this study, 32 patients (39.0%) showed procedure-related toxicities and 1 patient (1.2%) showed grade 3 hepatic subcapsular hemorrhage after the treatment and was treated with hepatoarterial embolization. In general, the adverse effects seen in 31 patients (37.8%) were of grade 1 or 2 and were transient, including RILD in 5 (6.1%), minor hemorrhage in 28 (34.1%), mild abdominal pain in 17 (20.7%), slight fatigue in 11 (13.4%), and slight pneumothorax in 4 (4.9%) (**Table 2**). No patients experienced complications of anorexia, nausea or vomiting, or diarrhea. Only one patient developed a hepatic abscess 3 months after the RSI; however, the symptoms were alleviated after medication. None of the patients in this study experienced RSI-related toxicities from radiation-induced liver disease.

**Table 2:** Complications experienced by the patients following treatment.

Complications	N	%
procedure-related toxicities		
No	50	61.0
Yes	32	39.0
Radiation-induced liver disease		
No	77	93.9
Grade 1	5	6.1
Hemorrhage		
No	53	64.6
Grade 1-2	28	34.1
Grade 3	1	1.2
Abdominal pain		
No	65	79.3
Grade 1	17	20.7
Fatigue		
No	71	86.6
Grade 1	11	13.4
Pneumothorax		
No	78	95.1
Grade 1	4	4.9

### Survival and Factors Affecting Outcomes

The median LPFS time was 16 months (95% confidence interval (CI) range, 12.6–19.4 months). The 1-, 3-, and 5-year LPFS rates were 63.8%, 27.1%, and 7.9%, respectively. The median liver PFS time was 15 months (95% confidence interval (CI) range, 11.9–18.1 months). The 1-, 3-, and 5-year liver PFS rates were 57.0%, 17.1%, and 6.0%, respectively. The median OS after the seed implantation was 23 months (95% CI, 15.2–30.8 months). The 1-, 3-, and 5-year OS rates were 74.8%, 32.9%, and 12.6%, respectively.

**Figure 2:**
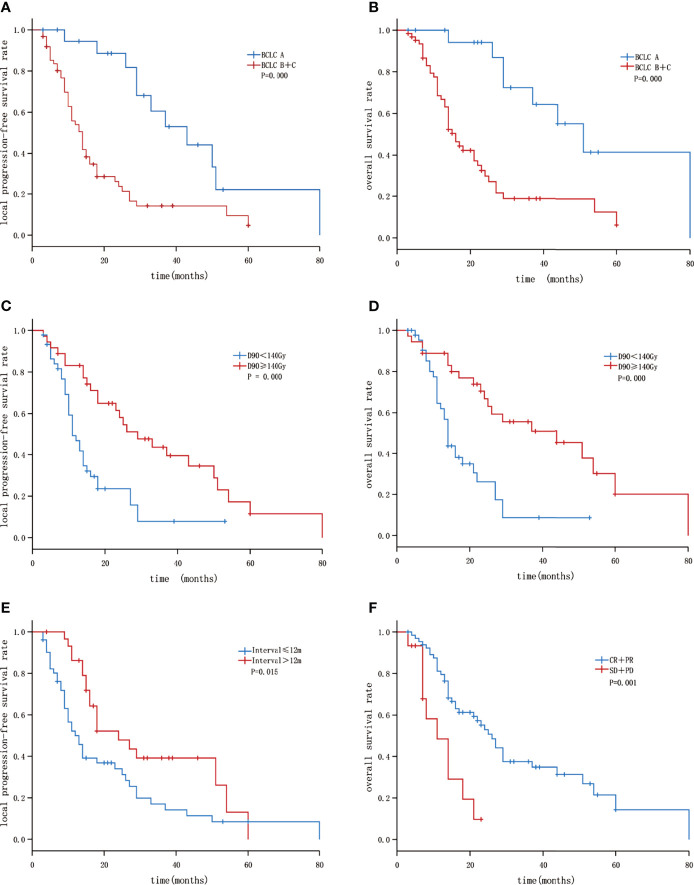
Kaplan–Meier curves showing LPFS and OS: **(A)** The LPFS of patients with BLCL A and B+C; **(B)** the OS of patients with BLCL A and B+C; **(C)** the LPFS of patients with GTV D90 <140 Gy and ≥140 Gy; **(D)** the overall survival of patients with GTV D90<140 Gy and ≥140 Gy; **(E)** the LPFS of patients with Interval ≤12 months and >12 months; **(F)** the OS of patients with short-term efficacy CR+PR and SD+PD.

Univariate analysis showed that a maximum tumor diameter of ≤ 4.5 cm, BCLC stage A, Interval time between the previous treatment and seed implantation > 12 months, and D90 ≥ 140 Gy were correlated with improved LPFS (P < 0.05 **Figure 1**). These four factors, age group, with or without extrahepatic metastasis, with or without portal vein tumor thrombus(P < 0.2) were included to Cox multivariate analysis. Multivariate analysis showed that the independent factors influencing the local control rate included the BCLC stage, Interval time, and D90 (P = 0.001, 0.001, and 0.046 respectively, **Table 3** and **Figure 2**).

**Table 3:** Univariate and multivariate analyses of factors influencing local progression-free survival.

Factor	n	Median (month)	1-year (%)	3-year (%)	5-year (%)	Univariate analyses		Multivariate analyses	
						*X^2^*	P-value	*HR* (95%*CI*)	P-value
Age(year)						2.572	0.109		
≤60 y	43	25	68.1	31.3	14.3				
>60 y	39	14	59.0	22.8	0				
Child-Pugh						0.392	0.531		
A	52	23	65.9	30.1	11.1				
B	30	14	60.0	21.2	7.1				
Extrahepatic metastasis						2.255	0.133		
No	52	23	69.6	31.8	14.9				
Yes	30	14	53.9	18.7	0				
portal vein tumor thrombus						3.364	0.067		
No	67	18	67.1	30.1	8.0				
Yes	15	10	48.2	12.1	0				
Tumor diameter						4.901	0.027		
≤4.5 cm	43	26	82.5	32.5	10.5				
>4.5 cm	36	11	44.6	19.6	9.8				
BCLC stage						13.412	0.000	0.28 (0.13~0.61)	0.001
A	20	43	89.4	61.1	22.3				
B+C	62	14	54.0	14.2	4.7				
interval time						5.897	0.015	2.59 (1.44~4.66)	0.001
≤12 m	52	13	50.0	17.0	8.5				
>12 m	30	24	86.2	47.9	13.1				
D90/Gy						13.061	0.000	1.83 (1.01~3.33)	0.046
<140	46	11	47.0	7.9	0				
≥140	36	29	83.2	43.9	11.5				

Univariate analysis showed that BCLC stage A, and D90 ≥ 140 Gy were correlated with a better liver PFS rate (P < 0.05). Interval time between the previous treatment and seed implantation > 12 months was correlated with improved liver PFS (P=0.057).Multivariate analysis showed that the independent factors influencing the liver PFS rate included BCLC stage and Interval time (P = 0.006, and 0.033 respectively).

Moreover, univariate analysis showed that age ≤ 60 years, absence of portal vein tumor thrombus, BCLC stage A, D90 ≥ 140 Gy, and short-term efficacy (in terms of CR + PR) were correlated with a better OS rate (P < 0.05). Multivariate analysis showed that the independent factors influencing the OS rate included BCLC stage, D90, and short-term efficacy (P = 0.008, 0.016, and 0.011 respectively, **Table 4** and **Figure 2**).

**Table 4:** Univariate and multivariate analyses of factors influencing overall survival.

Factor	n	Median (month)	1-year (%)	3-year (%)		5-year (%)	Univariate analyses		Multivariate analyses	
							*X^2^*	P-value	*HR* (95%*CI*)	P-value
Age(year)							4.392	0.036		
≤60 y	43	27	82.1		39.7	23.1				
>60 y	39	16	66.9		25.7	0				
portal vein tumor thrombus							6.661	0.010		
No	67	25	79.0		37.2	13.6		
Yes	15	13	54.2		12.6	0		
BCLC stage							11.738	0.001	0.31 (0.13~0.74)	0.008
A	20	51	100.0		72.4	41.4		
B+C	62	16	66.7		18.9	6.3		
D90/Gy							13.273	0.000	2.24 (1.16~4.32)	0.016
<140	46	14	62.0		8.7	0		
≥140	36	44	88.9		55.6	20.2		
Short-term Efficacy							12.083	0.001	0.38 (0.18~0.80)	0.011
CR+PR	67	26	79.5		37.5	14.3		
SD+PD	15	11	48.5		0	0		

**Figure 3:**
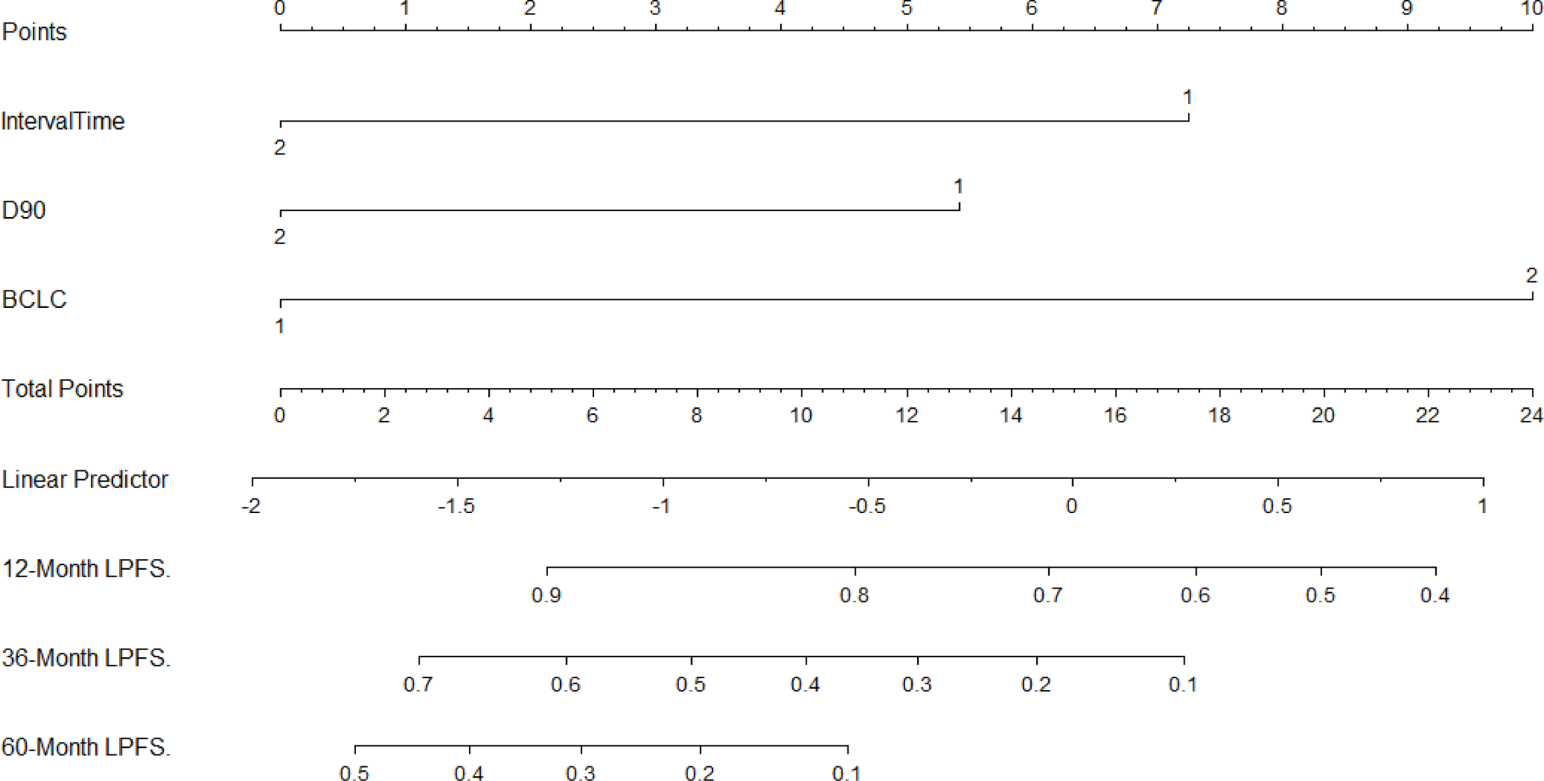
Nomogram to predict the local recurrence after seed implantation (For Interval: 1 = “≤12 months”, 2 = “>12 months”; For D90 (Gy): 1 = “< 140”, 2 =“≥140”; For BCLC: 1 = “A”, 2 =“B + C”).

**Figure 4:**
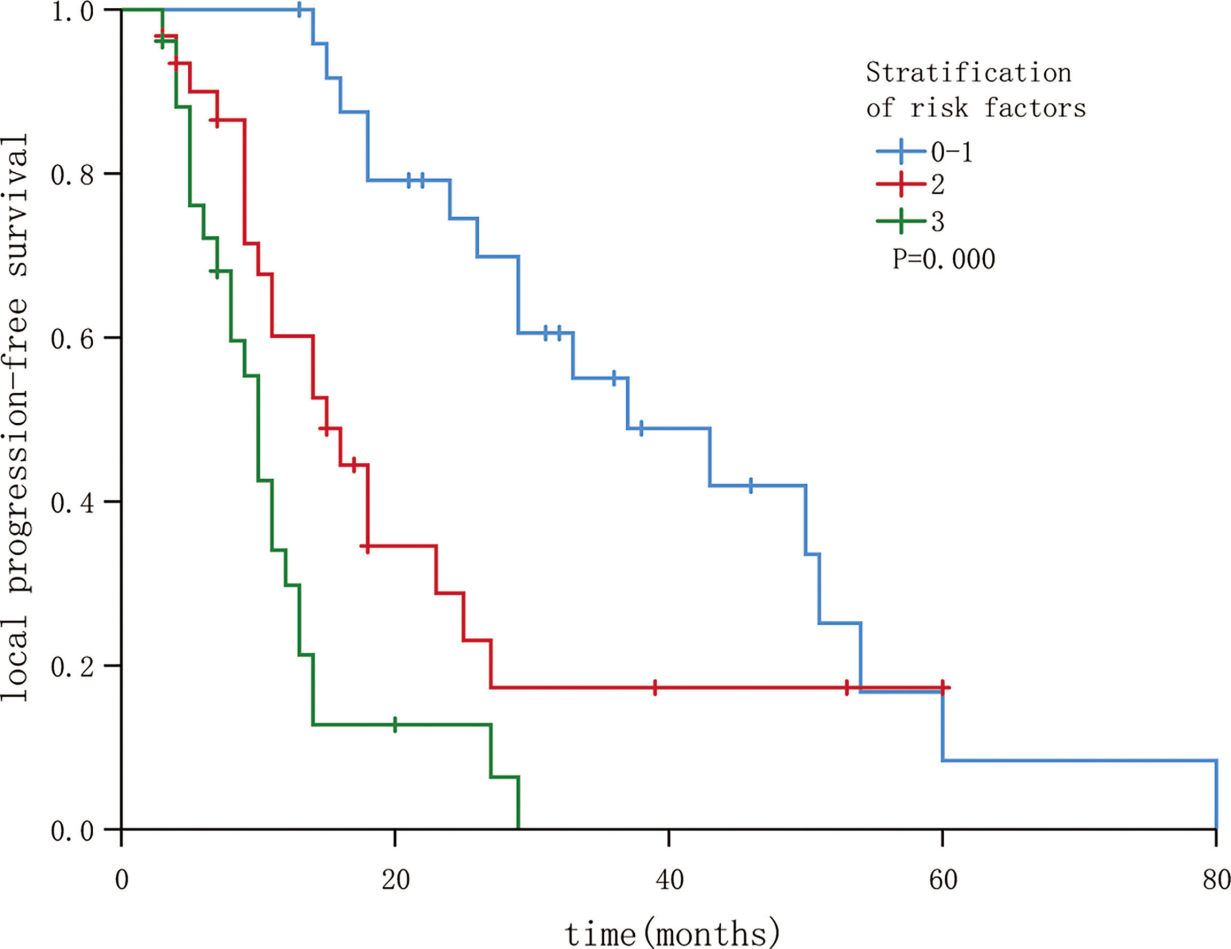
Local progression-free survival in patients following stratification by risk factors (P < 0.05).

The three independent factors influencing the LPFS rate, as identified *via* multivariate analysis, were used to plot nomograms (**Figure 3**), and the C-index (per internal validation) was 0.820 (95% CI, 0.767–0.873), suggesting good agreement between the projected and actual values. Independent risk factors for poor local control included an interval of ≤12 months between the previous treatment and seed implantation, D90 < 140 Gy, and BCLC stages B and C; significant differences were observed among patients with 0–1, 2, and 3 risk factors (P < 0.05). The 3-year LPFS rate reached 55.0% in patients with 0–1 risk factors (median LPFS, 37 months), whereas the median LPFS was 15 and 10 months for patients with 2 and 3 risk factors, respectively (**Figure 4**).

## Discussion

Patients who undergo hepatectomy and liver transplantation experience up to 40%–70% tumor recurrence within 5 years (15, 16). Incomplete radiofrequency ablation occurs due to large tumor size or lesions located adjacent to major vessels and the liver hilum. Locoregional recurrence and unsatisfactory survival in some patients is attributed to the incomplete ablation (17–22). According to the literature, 13.0% and 28.2% of HCC patients achieved pathological and radiographic CR after TACE (23, 24), and 43.2% experienced recurrence after radiographic CR (24).

External beam radiotherapy could be considered an alternative treatment option for rHCC (20). The ESMO guidelines consider selective internal radiation therapy as an option for patients with HCC (25). RSI, as permanent interstitial brachytherapy, is increasingly used in cancer treatment (9), including for such cancers as non-small cell lung cancer, residual hepatocellular carcinoma, and recurrent rectal cancer (26–28). Studies reporting the results of 64 patients with unresectable primary and metastatic liver tumors who underwent ^125^I brachytherapy between 1989 and 2002 showed that ^125^I brachytherapy was a safe and effective alternative technique that could facilitate long-term local control and increased survival (29). Studies indicated that ^125^I seed implantation for residual tumors following radiofrequency ablation (RFA) or TACE was associated with better local and intrahepatic tumor control, and improved long-term survival compared with those following RFA or TACE alone (30, 31). In a study by Song et al, where 57 patients with rHCC were enrolled and treated with CT-guided I-125 RSI, the median OS time was 23.6 months, and the 2- and 3-year survival rates were 46.1% and 24.3%, respectively (32). The survival rate observed in our study is comparable with that reported in this previous study (we reported a median OS time of 23 months and 2- and 3-year survival rates of 46.4% and 32.9%, respectively). Further, the median OS time was longer than that in the patients who received external beam radiotherapy (55 weeks) and sorafenib (43 weeks) in a previous study (33). These results indicate that CT-guided I-125 RSI is an effective treatment choice for recurrent and residual HCC.

Several studies have indicated various prognostic factors for advanced HCC, including the BCLC stage, Child-Pugh score, and implant volume (29–32). In this study, the BCLC stage, interval time between the previous treatment and seed implantation, and D90 were found to be independent factors influencing the local control rate.The BCLC stage, interval time were identified as factors influencing the liver PFS rate. And the BCLC stage, D90, and short-term efficacy were influencing the OS rate.

The BCLC system is the most widely used staging system for prognosis to determine the HCC stage, including the tumor burden, severity of liver disease, and patient’s performance status (34). In our study, BCLC stage A was correlated with a better LPFS, liver PFS and OS than stages B and C.

Xu et al. reported that the time to recurrence was the best determinant for prognosis in BCLC stage B or C for intrahepatic recurrence of HCC after resection, and the OS in the late-recurrence group (≥ 2 years) was significantly better than that in the early-recurrence group (< 2 years) (2). In our study, the time to recurrence was used to classify patients into the early-recurrence group (≤12 months) and the late-recurrence group (>12 months). The LPFS and liver PFS of the late-recurrence group (m-LPFS: 24 months, and m-liver PFS: 18 months) was better than that of the early-recurrence group (m-LPFS: 13 months, and m-liver PFS: 11 months).

A personalized dosimetry approach is accurate according to radiobiological rules, in which the threshold tumor absorbed radiation dose needs to be reached to achieve an effect. A randomized multicenter study of selective internal radiation therapy with yttrium-90-loaded glass microspheres in patients with HCC reported that patients who received a tumor dose of 205 Gy or higher had significantly a better objective response and OS (35). To date, there have been few studies on the dose of ^125^I radioactive seeds and local control of tumors. Ji et al. reported that the local control rate was higher with D90 ≥ 120 Gy than with D90 < 120 Gy for recurrent head and neck cancer following external beam radiotherapy (36). In our study, the cut-off value was 140 Gy. A high dose (D90 ≥ 140 Gy) was associated with better LPFS (29 months vs 11 months) and OS (44 months vs 14 months) than a dose of less than 140 Gy.

Short-term efficacy is one of the factors affecting the survival of HCC patients after treatment. Zhang et al. reported that short-term efficacy (in terms of CR and PR) influenced the local control and OS in patients who received CT-guided I-125 RSI for esophageal cancer with cervical lymph node metastasis after radiotherapy (37). Our study showed that patients with CR or PR had a better OS (26 months vs 11 months) than those with SD or PD.

In this study, we plotted LPFS nomograms with three dependent factors: BCLC stage, interval between the previous treatment and seed implantation, and D90. Patients with 0 or 1 risk factors showed better local control efficacy. The BCLC stage, D90, and short-term efficacy were related to survival after seed implantation. I-125 seed implantation with a sufficient radiation dose as local therapy had a favorable effect on LPFS and a further long-term influence on the survival after seed implantation with better short-term efficacy.

During the follow-up in our study, only 15.9% of patients were diagnosed with intrahepatic recurrence, whereas 30.5% experienced intrahepatic metastasis outside of the target tumors and 32.9% patients experienced extrahepatic distant metastasis after seed implantation. Because relapse outside target tumors is the major cause of treatment failure, RSI in combination with novel systemic therapies such as targeted therapies and/or immune checkpoint inhibitors could be a potential treatment strategy (4, 5).

All patients were successfully allocated the RSI schedule without any interruption caused by treatment-related toxicities. Only one patient showed a side effect (grade 3 hemorrhage) and was treated successfully with hepatoarterial embolization. No other severe adverse effects were observed.

3D-PCT guided RIS implantation could not only reduce the dosimetric differences between pre-and post-plan but also lower the difficulty of puncture (38, 39). In our multicenter study, 76 patients received free-hand guided I-125 seed implantation and 6 cases received 3D-PCT guided implantation. The two groups was not performed in statistical analysis, because of large different number of cases.

This multicenter retrospective study had some limitations. First, the population from different multicenter is heterogeneous of patient characteristics. In addition, we could not validly group patients with similar treatment histories. Therefore, a larger, prospective study is required to confirm the dose-response curve for I-125 RSI of rHCC.

## Conclusions

Iodine-125 seed implantation are well tolerated by patients with rHCC. And this therapy can allow substantial tumor control and can serve as a salvage treatment. The BCLC stage, interval, and D90 were found to influence the local control. In future investigations, BCLC stage A, interval time to recurrence >12 months could be the selection criteria for I-125 RSI treatment of patients with rHCC.

## Data Availability Statement

The original contributions presented in the study are included in the article/supplementary material. Further inquiries can be directed to the corresponding authors.

## Ethics Statement

Written informed consent was obtained from the individual(s) for the publication of any potentially identifiable images or data included in this article.

## Author Contributions

Guarantor of integrity of the entire study, KZ and JW. Study concepts and design, KZ and JW. Literature research, QY and LW. Clinical studies, YM, YS, CH, XH, CY, HH, KZ, and QY. Experimental studies/data analysis, QY, LW, YM, and CY. Statistical analysis, QY and BL. Manuscript preparation and editing, QY, LW and BL. All authors contributed to the article and approved the submitted version.

## Funding

This study was supported by the Shandong Provincial Medicine and Health of Social Development Project (202009030304). The funding sources had no role in the design, writing of the report, or decision to submit the paper for publication.

## Conflict of Interest

Author YM and YS are employed by Staff Hospital of Chengde Iron and Steel Group Co. Ltd.

The remaining authors declare that the research was conducted in the absence of any commercial or financial relationships that could be construed as a potential conflict of interest.

## Publisher’s Note

All claims expressed in this article are solely those of the authors and do not necessarily represent those of their affiliated organizations, or those of the publisher, the editors and the reviewers. Any product that may be evaluated in this article, or claim that may be made by its manufacturer, is not guaranteed or endorsed by the publisher.
